# Case Report: Rubella Virus-Induced Cutaneous Granulomas in Two Pediatric Patients With DNA Double Strand Breakage Repair Disorders – Outcome After Hematopoietic Stem Cell Transplantation

**DOI:** 10.3389/fimmu.2022.886540

**Published:** 2022-06-02

**Authors:** Ulrich Baumann, Johannes H. Schulte, Jonathan P. Groß, Rita Beier, Marius Ludwig, Volker Wahn, Jörg Hofmann, Britta Maecker-Kolhoff, Martin Sauer, Petra Kaiser-Labusch, Negin Karimian, Ulrike Blume-Peytavi, Franziska Ghoreschi, Hagen Ott, Ludmila Perelygina, Christian Klemann, Oliver Blankenstein, Horst von Bernuth, Renate Krüger

**Affiliations:** ^1^ Paediatric Pulmonology, Allergy and Neonatology, Hannover Medical School, Hannover, Germany; ^2^ Department of Pediatric Hematology, Oncology and Stem Cell Transplantation, Charité - Universitätsmedizin Berlin, corporate member of Freie Universität Berlin, Humboldt-Universität zu Berlin, and Berlin Institute of Health (BIH), Berlin, Germany; ^3^ Paediatric Oncology and Hematology, Hannover Medical School, Hannover, Germany; ^4^ Department of Pediatric Respiratory Medicine, Immunology and Intensive Care Medicine, Charité - Universitätsmedizin Berlin, corporate member of Freie Universität Berlin, Humboldt-Universität zu Berlin, and Berlin Institute of Health (BIH), Berlin, Germany; ^5^ Labor Berlin GmbH, Department of Virology, Berlin, Germany; ^6^ Institute of Virology, Charité – Universitätsmedizin Berlin, corporate member of Freie Universität Berlin and Humboldt-Universität zu Berlin, Berlin, Germany; ^7^ Prof. Hess Children’s Hospital, Klinikum Bremen-Mitte, Bremen, Germany; ^8^ Department of Dermatology and Allergology, Charité-Universitätsmedizin, corporate member of Freie Universität Berlin, Humboldt-Universität zu Berlin, and Berlin Institute of Health (BIH), Berlin, Germany; ^9^ Department of Paediatric Dermatology and Allergology, Center for Rare Congenital Skin Diseases, Children’s Hospital Auf der Bult, Hannover, Germany; ^10^ Division of Viral Diseases, Centers for Disease Control and Prevention, Atlanta, GA, United States; ^11^ Institute for Experimental Pediatric Endocrinology, Newborn Screening Laboratory, Charité - Universitätsmedizin Berlin, corporate member of Freie Universität Berlin, Humboldt-Universität zu Berlin, and Berlin Institute of Health (BIH), Berlin, Berlin, Germany; ^12^ Berlin Institute of Health at Charité – Universitätsmedizin Berlin, Berlin, Germany; ^13^ Charité - Universitätsmedizin Berlin, corporate member of Freie Universität Berlin, Humboldt-Universität zu Berlin, and Berlin Institute of Health (BIH), Berlin-Brandenburg Center for Regenerative Therapies (BCRT), Berlin, Germany; ^14^ Labor Berlin GmbH, Department of Immunology, Berlin, Germany

**Keywords:** inborn errors of immunity, primary immunodeficiency, Ataxia telangiectasia, Artemis deficiency, rubella virus vaccine strain, granuloma formation, stem cell transplantation

## Abstract

We report two patients with DNA repair disorders (Artemis deficiency, Ataxia telangiectasia) with destructive skin granulomas, presumably triggered by live-attenuated rubella vaccinations. Both patients showed reduced naïve T cells. Rapid resolution of skin lesions was observed following hematopoietic stem cell transplantation. However, the patient with AT died due to complications of severe hepatic veno-occlusive disease 6 month after HSCT. Dried blood spots obtained after birth were available from this patient and showed absent T-cell receptor excision circles (TRECs). Therefore, newborn screening may help to prevent patients with moderate T-cell deficiency from receiving live-attenuated rubella vaccine potentially causing granulomas.

## Introduction

Vaccine rubella virus (RuV)-induced granuloma (RG) was first described in patients with combined immunodeficiency (CID) in 2014 (1). Initially, patients with DNA double-strand breakage repair disorders were reported. To date, a growing number of patients with inborn errors of immunity (IEI) of different genetic origin and vaccine RuV-induced granulomas have been published (2), also reviewed in (3). Sustained resolution of RG has only been observed after hematopoetic stem cell transplantation (HSCT) (4). However, despite reduced-intensity conditioning, HSCT in patients with DNA repair disorders is complicated by higher transplant-associated morbidity and mortality as compared to other IEI patients (5). In the current issue of Frontiers in Immunology, Perelygina et al. discuss pathomechanisms, clinical characteristics and treatment of vaccine RuV-induced granulomas in a cohort of 28 patients with various IEI with CID (3). HSCT has been shown to be a therapeutic option, but 2 out of 7 patients were lost due to transplantation related mortality. Here, we report two children with DNA repair disorders and destructive skin RG that showed prompt resolution following HSCT. However, one patient, also described in the cohort (Pat. 28), died from HSCT complications, increasing the mortality to 3 out of 7 transplanted patients.

## Case Reports

**Figure 1:**
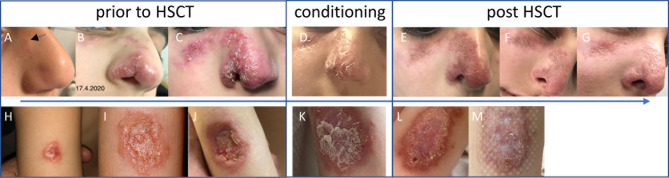
Skin lesion prior to HSCT **(A–C, H–J)**, at conditioning **(D, K)**, and post-HSCT **(E–G, L, M)** of patient 1 (upper panel) and patient 2 (lower panel). Pictures of patient 1 were taken at age 8.5 **(A)**, 9.3 **(B)** and 10.1 **(C)** years, and day +4 **(E)**, +18 **(F)**, and +36 **(G)** after HSCT. Pictures of patient 2 were taken at age 4.7 **(H)**, 5.6 **(I)**, 5.7 **(J)** years, at conditioning **(K)**, and at day +5 **(L)**, and +15 **(M)** after HSCT.

Patient 1: This patient suffered from recurrent diarrhea and chronic unproductive cough beginning in infancy. At school age, he developed a productive cough with bronchiectasis, vitiligo, and retarded growth. Immunological workup at preschool age had shown moderate CD4+ and CD8+ T-cell lymphocytopenia, normal immunoglobulin isotype levels and vaccine-induced antibodies. Next-generation sequencing covering a panel of 104 genes yielded no pathological findings at the age of 6.5 years. Two years later, he suffered from pneumonia due to varicella-zoster virus with a prolonged course of over 2 months. He was subsequently commenced on immunoglobulin substitution. At the age of 7.5 years, granulomatous lesions appeared at the heel and the nose (**Figure 1A**). Skin biopsy of the granulomatous lesions showed rubella antigen in epitheloid cells, but neither in CD15+ granulocytes nor CD68+ macrophages. No testing for specific rubella strains was performed. The boy had received 2 MMR live vaccinations in his 4^th^ year of life. Topical steroid application was not effective in limiting progression. At age 8.5 years, whole-exome sequencing revealed a homozygous splice variant in *DCLRE1C (c.464+1G>A*), suggestive for a DNA repair disorder with combined immunodeficiency consistent with Artemis deficiency. As the patient was in a stable condition receiving immunoglobulin treatment, the parents were reluctant to agree to a proposed HSCT. It was due to progressive and disfiguring destruction of the right nostril (**Figure 1**) that the family eventually accepted HSCT. He subsequently received HSCT from a matched related donor at the age of 10.3 years. Conditioning was performed according to protocol C of the IEWP guideline for hypomorphic Artemis SCID (6) including busulfan (AUC 65 mg*h/l), fludarabine (4 x 45mg/m2/d) and anti-thymocyte globulin (3 x 10mg/kg/d). GvHD prophylaxis consisted of cyclosporin A and mycophenolate mofetil. Engraftment was achieved as expected with a bone marrow graft (leukocytes >1/nl and neutrophils >0.5/nl on day 26, platelets >50/nl on day 34). The post-HSCT course was complicated by an intercurrent secondary von Willebrand Jürgens syndrome and by BK-viremia which was successfully treated with cidofovir. 1.5 years post-HSCT, the patient developed self-limiting autoimmune thrombocytopenia which was more pronounced during viral upper airway infections. Chimerism after HSCT was mixed at day +16 (84% donor) and subsequently always 100% donor. The cutaneous lesions had already started to improve under conditioning and healed largely within two months after HSCT. Even the deep nasal defect partially closed. The lesions before and after HSCT are shown in **Figures 1E–G** clinical characteristics are summarized in **Table 1**. Unfortunately, the Guthrie card of patient one was not available for TREC-screening.

Patient 2: A five-year-old microcephalic girl [patient 28 in (3)] was diagnosed with Ataxia telangiectasia (AT) at the age of 3 years following progressive gait disturbances, recurrent viral and bacterial airway infections and failure to thrive (compound heterozygous mutations in *ATM:* c.2098C>T, p.Gln700*; second mutation probably intronic). She had also suffered from a progressive granulomatous skin lesion on the left upper arm since the age of three years (**Figures 1H–J**). The immunologic evaluation showed a deficiency of IgA, IgG_2_, IgG_4_ and pneumococcal antibodies with normal total IgG and normal antibodies to tetanus-toxoid. Naïve CD4+ T cells were repeatedly markedly reduced with normal lymphocyte, total B- and T-cell counts at initial evaluation (**Table 1**). Evaluation of the stored dried blood spot (DBS) obtained from the neonatal screening sample showed no TREC copies by using a standardized newborn screening rt-PCR assay (ImmunoIVD, Nacka Strand, Sweden). Prior to the diagnosis of AT the girl had received live vaccines against MMR at the age of 12 and 14 months. Skin biopsy of the lesion at the age of 5 years revealed a granulomatous inflammation, RT-PCR and immunohistochemistry from biopsy material showed rubella vaccine strain RA27/3 (3). The lesion did not improve upon treatment with local corticosteroids or immunoglobulin substitution (initiated due to specific antibody deficiency). HSCT was offered as a therapeutic option once local destructive growth of the lesion (**Figure 1J**) with increasing pain and pruritus occurred. The parents were counseled by experts for HSCT in IEI from two different hospitals. Limited data on HSCT outcome in patients with AT and risks were intensely discussed. The parents were aware that HSCT would presumably not affect progression of ataxia. They were also given the opportunity to contact parents of AT patients that had been transplanted.

**Table 1:** Clinical, genetic and immunological characteristics of patient 1 and 2.

	Reference values	Patient 1	Patient 2
**IEI diagnosis**		ARTEMIS	AT
**Age at blood sampling**		6 y 7 m	5 y 8 m
**CD3 [/µl]**	700-4200	798	820
**CD4 [/µl]**	300-2000	474	460
**CD8 [/µl]**	300-1800	**239**	**270**
**Naive CD4 [% CD4; /µl]**	> 25%	**13%**; **63**	**4%**; **18**
**γ/δ T cells (%)**	< 10	9.9	**12**
**B cells [/µl]**	200-1600	**183**	400
**NK cells [/µl]**	90-900	112	**1270**
**IgG [g/l]**	5.04-14.64	6.74*	10.13*
**IgA [g/l]**	0.27-1.95	**< 0.01**	**< 0.1**
**IgM [g/l]**	0.52-1.90	1.76	1.24
**IgE [IE/ml]**	5-59 iE/ml	< 5	<5
**IgG1 [g/l]**	3.0-8.4	4.71*	7.17*
**IgG2 [g/l]**	0.70-2.55	1.49*	0.96*
**IgG3 [g/l]**	0.170-0.970	0.28*	0.198*
**IgG4 [g/l]**	0.017-1.157	< 0.01*	0.084*
**Anti-Tetanus-IgG [iU/ml]**	≥ 0.1	0.15*	> 7.00*
**Anti-Pneumococcal-IgG**		12 mg/l	< detection limit*
**Anti-Rubella IgG [U/ml]**		89, IgM neg.	261, IgM negative
**BG IgM-Isoaggl.Titer**		n.d. / **1:2**	1:16/1:32
**Infections**		VZV pneumonia	Upper and lower airway infections
**Autoimmune manifestations**		vitiligo	none

*prior to IgG-substitution.

in bold: values above or below reference range.

HSCT (TCRαβ/CD19-depleted peripheral blood stem cells of a matched (10/10) unrelated donor with add-back of 1 x 10^6^ T cells/kg body weight) was performed at the age of 6 years. To prevent excessive toxicity and to reduce the risk of malignancy in DNA repair disorders, a reduced-intensity conditioning (RIC) was applied consisting of fludarabine (5 x 30 mg/m^2^/d), cyclophosphamide (4 x 30 mg/kg/d) and rabbit anti-thymocyte globulin (ATG Fresenius^®^ 3 x 20 mg/kg/d) derived from Bakhtiar et al. (7). The skin lesion`s regression was already observed upon treatment with fludarabine and cyclophosphamide prior to HSCT. The girl received standard GvHD-prophylaxis with ATG, mycophenolate mofetil and cyclosporine A. The patient showed timely engraftment with platelets >100/nl on day 8, leucocytes >1/nl on day 12, neutrophils >0.5/nl on day 14, and >1/nl on day 16 after single stimulation with granulocyte-colony stimulating factor on day 15. Whole blood chimerism after HSCT was mixed at day +40 (73% donor) and increased to 100% donor five months post HSCT. She developed a grade III graft-versus-host disease of the skin on day 21, which was treated successfully with topical tacrolimus and corticosteroids as well as systemic corticosteroids. After HSCT, a reactivation of adenovirus in the stool and the blood as well as EBV in the blood was observed with low copy numbers and no severe clinical complications without antiviral therapy. The granuloma resolved with scarring within 10 weeks following HSCT (**Figures 1L, M**, no pictures were taken after day 15), the first signs of resolution were already seen during conditioning. However, HSCT was complicated by severe and therapy-refractory hepatic veno-occlusive disease (VOD). Sadly, the girl died from complications of VOD 6 months after HSCT [after submission of (3)].

## Discussion

Our report demonstrates that normal ranges for total IgG levels, IgG vaccine responses to protein antigens, total lymphocyte as well as total T- and B-cell counts cannot exclude CID. Delayed diagnosis of CID in patient 1 was complicated by disfiguring rubella-associated skin granuloma. The report underscores the effectiveness of HSCT as, to date, the only known curative treatment of RG in patients with IEI. Granulomas of both patients started to improve within days after initiation of conditioning and largely resolved (with scarring in patient 2) within the first months after HSCT. Even disfiguring lesions in patient 1 healed to a major extent. Resolution of granulomas starting during conditioning and completing by several weeks following HSCT suggests that healing of RG largely depends on patients` depletion of rubella-harboring neutrophils and monocytes/macrophages (see Ref. 3) rather than on B- and T-cell immune reconstitution.

However, patient 2 [patient 28 in (3)] died 6 months after HSCT due to complications of severe hepatic VOD, thus increasing the number of patients in the cohort who died following transplantation to 3 out of 7. Only 7 out of 28 patients in the cohort underwent HSCT, possibly explained by lack of knowledge on treatment of RG in the past. The low number may also reflect concerns about the high morbidity and mortality of HSCT in high-risk patients. No other patient with AT described in the cohort (n=4) underwent HSCT. HSCT in DNA double-strand repair disorders has been reviewed recently (5), indicating particularly poor outcome in patients with AT. Reduced conditioning regimens and adapted protocols for AT appear to improve outcomes (8), the impact on neurologic deterioration in AT patients has to be determined. Other therapeutic options with better outcomes need to be developed.

RG can occur weeks to decades following infection or vaccination [reviewed in (3)], thus it is unclear how many yet unaffected patients with CID/DNA repair disorders will develop RG later in life. Early sensitive and specific risk factors for RG to avoid MMR live-vaccines in infants are yet to be identified. Interestingly, in a cohort of AT, 7 out of 8 patients with granulomas showed IgA deficiency (9). In addition, IgA deficiency has been identified as a surrogate marker for immunodeficiency and mortality in AT patients (10). However, transient IgA deficiency is also common in healthy infants and thus not useful for risk assessment prior to MMR vaccination in patients with AT.

Live vaccines (rotavirus, varicella, measles, rubella, yellow fever, BCG) should be strictly avoided in infants with (S)CID and further defined IEI, since severe vaccine-related infections may occur (11, 12). However, MMR is a crucial live vaccine and avoidance will put children with IEI at risk of severe disease unless IgG substitution is performed. In patients with DNA repair disorders and CID, diagnosis of IEI is often delayed (13), with live vaccines commonly given prior to established IEI diagnosis. Thus, avoidance of rubella vaccination to prevent RG in children at risk is difficult. It is likely, that newborn screening (NBS) for SCID will help to identify some patients with non-SCID IEI at risk for RG as demonstrated for patient 2. However, it remains unclear whether patients identified by NBS as having moderately reduced TREC numbers not requiring HSCT may develop RG upon live vaccination. Further studies are needed to establish immunological parameters that allow the safe use of live vaccines in these infants. Alternatively, the development of a safe, non-live-rubella vaccine, either subunit or mRNA-based, should be considered.

## Data Availability Statement

The original contributions presented in the study are included in the article/supplementary material. Further inquiries can be directed to the corresponding author.

## Ethics Statement

Written informed consent was obtained from the minor(s)’ legal guardian/next of kin for the publication of any potentially identifiable images or data included in this article.

## Author Contributions

UB, JS, JG, ML, VW, JH, NK, UB-P, FG, LP, OB, HB and RK contributed patient data. UB, HB and RK wrote the manuscript. All coauthors read and approved the final version of the manuscript. All authors contributed to the article and approved the submitted version.

## Author Disclaimer

The findings and conclusions in this report are those of the authors and do not necessarily represent the official position of the United States Centers for Disease Control and Prevention.

## Conflict of Interest

Authors JH and HB were employed by Labor Berlin GmbH

The remaining authors declare that the research was conducted in the absence of any commercial or financial relationships that could be construed as a potential conflict of interest.

## Publisher’s Note

All claims expressed in this article are solely those of the authors and do not necessarily represent those of their affiliated organizations, or those of the publisher, the editors and the reviewers. Any product that may be evaluated in this article, or claim that may be made by its manufacturer, is not guaranteed or endorsed by the publisher.

## Abbreviations

AT, Ataxia telangiectasia; ATG, Anti-thymocyte globulin; CID, Combined immunodeficiency; DBS, Dried blood spot; EBV, Epstein Barr virus; GvHD, Graft versus host disease; HSCT, Hematopoietic stem cell transplantation; IEI, Inborn errors of immunity; MMR, Measles, mumps and rubella; NBS, Newborn screening; RG, Rubella-induced granuloma; RT-PCR, Reverse transcription polymerase chain reaction; SCID, Severe combined immunodeficiency; TREC, T-cell receptor excision circles; TRM, Transplantation related mortality;VOD, Veno-occlusive disease.
